# SIRT1通过调节Noxa表达影响非小细胞肺癌细胞株A549对顺铂的敏感性

**DOI:** 10.3779/j.issn.1009-3419.2016.02.01

**Published:** 2016-02-20

**Authors:** 彬 曹, 晓峰 何, 文公 王, 敏科 史

**Affiliations:** 210008 南京，南京大学医学院附属鼓楼医院胸心外科 Department of Thoracic-Cardio Surgery, the Affiliated Drum Tower Hospital of Nangjing University Medical School, Nanjing 210008, China

**Keywords:** 肺肿瘤, SIRT1, NOXA, 顺铂, 化疗, Lung neoplasms, SIRT1, NOXA, Chemotherapy, Cisplantin

## Abstract

**背景与目的:**

非小细胞肺癌的顺铂耐药是常见的临床现象，严重制约了患者的化疗效果，是亟待解决的问题。SIRT1和Noxa的表达变化影响肿瘤细胞对化疗药物的敏感性。本研究旨在研究SIRT1表达对非小细胞肺癌对顺铂的敏感性的影响，并探讨其涉及Noxa表达的机制，以求为提高非小细胞肺癌细胞对顺铂敏感性提供希望。

**方法:**

利用实时荧光定量PCR和Western blot分析A549细胞及顺铂耐药的A549/DDP细胞SIRT1及Noxa mRNA和蛋白水平的表达差异。利用siRNA干扰技术抑制A549/DDP细胞的SIRT1表达，进而使用Cell Titer Blue试验、流式细胞术从细胞增殖、细胞周期和细胞凋亡方面分析SIRT1沉默对A549/DPP细胞顺铂敏感性的影响。同时利用实时荧光定量PCR和Western blot分析SIRT1抑制对A549/DPP细胞Noxa表达的影响。

**结果:**

A549细胞和A549/DDP细胞对顺铂的敏感性有显著差异，与A549细胞相比，A549/DDP细胞的SIRT1表达较高，但Noxa表达较低。使用siRNA抑制A549/DPP细胞的SIRT1表达后，与未抑制SIRT1细胞相比，4 μg/mL顺铂处理后的细胞存活率降低，G2期/M期阻滞比例增加，凋亡率提高。同时，SIRT1沉默导致A549/DPP细胞的Noxa表达增加。

**结论:**

较高的SIRT1可能引起A549细胞对顺铂的耐药性，抑制SIRT1可以提高A549/DDP细胞对顺铂的敏感性，其机制可能涉及SIRT1对Noxa的调节。

## Abstract (en)

**Background and objective:**

The resistance of non-small cell lung cancer cells to cisplant is a common clinical phenomenon which could induce a poor therapeutic effect and should be difficult problem to be solved. SIRT1 and Noxa expression are associated with the chemotherapy for tumors. The present study focused on how SIRT1 expression influence the senstivity of non-small cell lung cancer cells and dissected the potential mechanism involved with Noxa.

**Methods:**

The difference of SIRT1 and Noxa expression between A549 cells and A549/DDP cells was detected by real-time quantitative PCR (qRT-PCR) and Western blot. SIRT1 targeted siRNA was uesed to inhibit the SIRT1 expression in A549/DDP, after transfection, Cell Titer Blue assay, flow cytometry were performed to analyze the cell viability, cell cycle and cell apoptosis in order to reveal the effect of inhibition of SIRT1 on sensitivity of A549/DDP cells to cisplant. Moreover, the expression changes of Noxa in A549/DDP cells after siRNA treatment were detected by qRT-PCR and Western blot.

**Results:**

There was a significant difference in senstivity to cisplant between A549 and A549/DDP cells. Compared with A549 cells, the A549/DDP cells showed a higher SIRT1 expression and lower Noxa expression. After transfected with SIRT1 targeted siRNA, the cell viability decreased accompanied with a increasing apoptosis rate, meanwhile, higher percent of G2/M phase was detected after the 4 μg/mL cisplant treatment. Further more, inhibition of SIRT1 could induce the Noxa expression in A549/DDP cells.

**Conclusion:**

Higher SIRT1 expression may induce resistance to cisplant in A549 cells. SIRT1 inhibition may improve the sensitivity of A549/DDP cells to cisplantin though modulating the Noxa expression.

顺铂以及基于顺铂的联合化疗方案已经成为了目前临床上治疗非小细胞肺癌（non-small cell lung cancer, NSCLC）的成熟方案，其效果明确，不良反应相对较小，同时费用经济^[1]^。但是，一些患者因为个体差异，对顺铂治疗不敏感或在治疗中产生了耐药性，导致了顺铂治疗方案的失败。因此，改善患者对顺铂的敏感性是临床亟待解决的问题，然而当前NSCLC患者对顺铂敏感性的差异机制并不十分明确^[2]^。SIRT1是一种依赖烟酰胺腺嘌呤二核苷酸的组蛋白去乙酰化酶，具有调节DNA损伤修复，调控细胞周期及凋亡的作用^[3]^。一些研究已经指出，在肿瘤细胞中SIRT1具有抗凋亡作用并与肿瘤细胞对化疗药物的敏感性降低有关。已有研究^[4]^发现对化疗耐受的肝癌患者SIRT1表达较高且预后较差。但是SIRT1对于NSCLC顺铂敏感性的影响和机制仍未得知。Noxa是Bcl-2家族的成员之一，具有凋亡诱导作用。在化疗药物的处理下，Noxa会出现过量表达，因此可能影响肿瘤细胞对化疗药物的敏感性。如有研究指出过低的Noxa表达会降低Bcl-2抑制剂ABT-737对食管癌细胞的敏感性^[5]^。本研究探讨了SIRT1对NSCLC细胞株A549顺铂敏感性的影响并分析了其对Noxa表达的调节以期揭示其机制。

## 1 材料和方法

### 1.1 主要试剂及仪器

A549细胞株（上海中国科学院细胞库）；顺铂（美国Sigma-Aldrich公司）；CellTiter-Blue分析试剂盒[普洛麦格（北京）生物技术有限公司]；荧光分光光度计；Trizol试剂（美国Invitrogen公司）；用ImProm-Ⅱ^®^反转录试剂盒[普洛麦格（北京）生物技术有限公司]；GO Taq^®^ qPCR Master Mix试剂盒[普洛麦格（北京）生物技术有限公司]；实时荧光定量PCR引物[宝生物工程（大连）有限公司]；siRNA序列（上海吉玛制药技术有限公司）；RIPA裂解液（上海碧云天生物技术有限公司）；小鼠抗人SIRT1多克隆抗体；小鼠抗人Noxa多克隆抗体；小鼠抗人β-actin多克隆抗体；辣根过氧化物酶标记的山羊抗小鼠二抗均购自美国Santa Cruz Biotechnology公司；ECL发光试剂盒（上海碧云天生物技术有限公司）；Lipofectamine^®^ 2000试剂盒美国Thermo Fisher Scientific公司）；Annexin V-FITC-PI双染凋亡分析试剂盒（美国Sigma-Aldrich公司）；细胞培养箱（美国Thermo Fisher Scientific公司）；BD FACSCalibur流式细胞仪（美国Beckman Coulter公司）；BrdU细胞周期分析试剂盒（美国BD公司）；BMG Ω荧光读板机（德国BMG LABTECH公司）。

### 1.2 细胞培养

A549细胞培养于含10%胎牛血清的DMEM培养基（美国GIBCO公司），培养环境为5%CO_2_、37 ℃。A549/DDP细胞培养环境同A549细胞，但培养基含2 μg/mL顺铂，以维持其耐药性。0.25%胰酶常规消化，选择生长良好的对数期细胞进行实验。

### 1.3 Cell Titer Blue试验分析A549细胞和A549/DDP细胞对顺铂的敏感性差异

将A549细胞和A549/DDP细胞以1×10^4^/孔的密度种植于96孔板。常规培养24 h后经行处理。以未经顺铂处理的细胞为对照组，其余细胞分别接受0.25 μg/mL、0.5 μg/mL、1.0 μg/mL、2.0 μg/mL、4.0 μg/mL、8.0 μg/mL、16.0 μg/mL梯度浓度的顺铂处理24 h，同时设立无细胞的培养孔作为空白调零组，每组设5个平行孔。24 h后，弃去孔中原有液体，加入72 μL Cell Titer Blue工作液（Cell Titer Blue试剂：DMEM=1:10）。继续孵育1 h后使用荧光读板机检测各组荧光强度并以公式存活率＝（处理组荧光强度/对照组荧光强度）×100%计算细胞存活率，通过曲线拟合计算半抑制浓度（half maximal inhibitory concentration, IC_50_）。

### 1.4 实时荧光定量PCR检测SIRT1和NOXA的mRNA表达

Trozal试剂提取A549细胞和A549/DDP细胞的总RNA。使用ImProm-Ⅱ^®^反转录试剂盒对提取出的总RNA进行反转录，以合成cDNA。反转录体系参照试剂盒提供的说明书。使用GO Taq^®^ qPCR Master Mix试剂盒对上述合成的cDNA进行PCR扩增。SIRT1的上游引物为：5’ GCC TCA TCT GCA TTT TGA TG’3，下游引物为：5’ TCT GGC ATG TCC CAC TAT CA’3；NOXA的上游引物为：5’TTC GTG TTC AGC TCG CGT CC’3，下游引物为：5’CTC GGT GTA GCC TTC TTG CC’3；内参选择β-actin，其上游引物为：5’CTC CAT CCT GGC CTC GCT GT’3，下游引物为：5’GCT GTC ACC TTC ACC GTT CC’3。反应体系和方案参照试剂盒说明书。使用2^-△△CT^法分析两种细胞的SIRT1和NOXA mRNA相对表达差异^[6]^。

### 1.5 Western blot检测SIRT1和NOXA的蛋白表达

常规培养A549细胞和A549/DDP细胞，RIPA裂解液提取总蛋白样品，8%的聚丙烯酰胺凝胶进行电泳并电转于PVDF膜，5%脱脂奶粉室温封闭1 h。分别使用1:1, 500的小鼠抗人SIRT1多克隆抗体、1:500小鼠抗人Noxa多克隆抗体以及1:5, 000鼠抗人β-actin多克隆抗体，4 ℃孵育12 h。接着使用PBST洗涤3次，除去未结合的一抗。使用辣根过氧化物酶标记的山羊抗小鼠二抗（1:1, 000）室温下孵育1 h-2 h，ECL发光试剂盒显色并分析条带^[7]^。

### 1.6 SiRNA转染降低SIRT1表达

针对SIRT1的特异性siRNA序列由上海吉玛制药技术有限公司设计并合成，正向序列为GCA AUA GGC CUC UUA AUU Att，反向序列为UAA UUA AGG CCU AUU GCtt。A549/DDP细胞以1×10^5^/孔的密度种植于24孔板，当细胞汇合度达70%时进行转染。转染使用Lipofectamine^®^ 2000试剂盒，同时设无关序列siRNA阴性转染对照（siRNA negative control, siRNA NC）。转染48 h后实时荧光定量PCR进行转染效果的验证。

### 1.7 降低SIRT1对A549/DDP细胞顺铂敏感性的影响

选择成功转染SIRT1 siRNA、无关序列siRNA及未经任何转染的A549/DDP细胞以1×10^5^/孔的密度种植于6孔板。细胞分为3组：以未经转染处理细胞为对照组（control）、转染SIRT1 siRNA组（SIRT siRNA）以及转染无关序列siRNA的阴性转染组（siRNA NC）。各组设两个亚组：4 μg/mL顺铂处理亚组（处理24 h）及未经顺铂处理亚组，每个亚组设5个平行孔。使用1.3提及的Cell Titer Blue试验分析各组细胞的存活率。

### 1.8 细胞凋亡分析

细胞分组及处理同1.7所述。收集处理后的细胞，70%预冷酒精固定细胞过夜，加入Annexin V-FITC和碘化丙啶避光孵育30 min，使用BD FACSCalibur流式细胞仪分析各组细胞凋亡情况。

### 1.9 细胞周期分析

细胞分组及处理同1.7所述。收集处理后的细胞，70%预冷酒精固定细胞过夜，加入BrdU和7-amino-actinomycin D（7-ADD）染液孵育20 min。使用BD FACSCalibur流式细胞仪分析各组细胞的细胞周期情况

### 1.10 降低SIRT1表达对Noxa表达的影响

细胞分组及处理同1.7所述。使用1.4及1.5中提到的方法分析各组细胞Noxa的mRNA和蛋白表达水平。

### 1.11 统计学方法

采用SPSS 17.0软件，数据以Mean±SD表示，两组间的比较采用*t*检验，多组间的比较采用单因素方差分析，多组间的两两比较采用*LSD*-*t*检验，以*P* < 0.05为差异有统计学意义。

## 2 结果

### 2.1 A549细胞和A549/DDP细胞对顺铂的敏感性差异

Cell Titer Blue分析可见549/DDP细胞对顺铂具有一定的耐药性（图 1）。顺铂处理A549细胞株的IC_50_为4.32 μg/mL，而处理A549/DDP的IC_50_为14.26 μg/mL。

**1:**
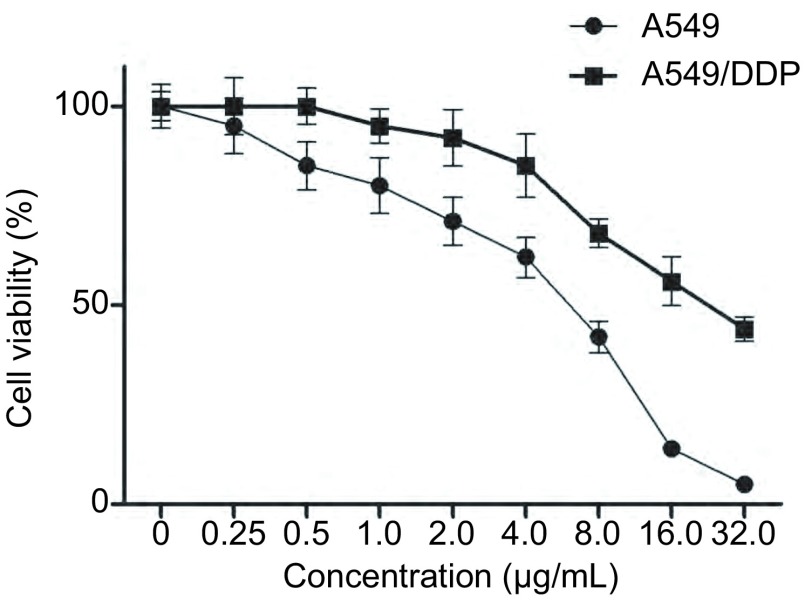
不同浓度顺铂处理后A549细胞和A549/DDP细胞的存活率 The cells viability of A549 and A549/DDP cells after treated with different concentration of cisplatin

### 2.2 A549细胞和A549/DDP细胞的SIRT1和Noxa表达存在差异

qRT-PCR分析显示，与A549细胞相比，A549/DDP细胞的SIRT1 mRNA水平较高，相对A549细胞的表达倍数为（2.51±0.42）（*P* < 0.05），但Noxa mRNA水平较低，相对A549细胞的表达倍数为（0.49±0.09）（*P* < 0.05）。Western blot结果qRT-PCR结果类似，提示A549/DDP细胞的SIRT1蛋白表达较高，但Noxa蛋白表达较低（图 2）。

**2:**
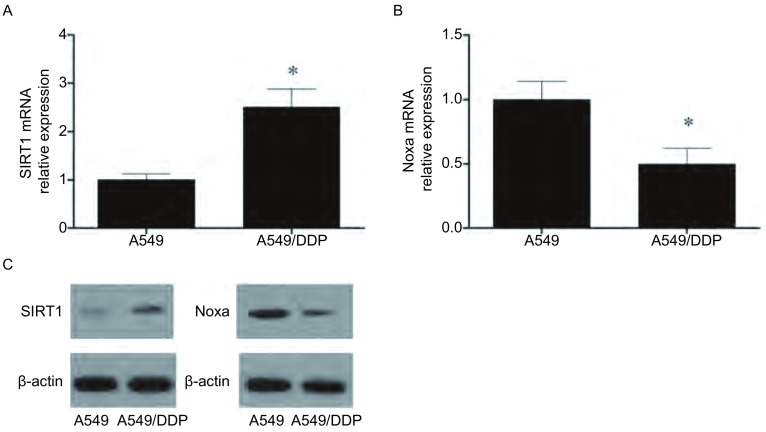
A549细胞A549/DDP细胞SIRT1及Noxa的表达差异。A：SIRT1 mRNA表达；B：Noxa mRNA表达；C：SIRT1蛋白表达。*：与A549细胞相比，*P* < 0.05。 The SIRT1 and Noxa mRNA expression difference between A549 and A549/DDP cells. A: IRT1 mRNA expression; B: Noxa mRNA expression; C: SIRT1 protein expression.*: indicate *P* < 0.05 compared with A549 cells.

### 2.3 降低SIRT1表达可以增强顺铂造成的细胞增殖抑制

如图 3所示，SIRT1转染可有效降低A549/DDP细胞的SIRT1 mRNA表达。针对SIRT1的siRNA转染可增加A549/DDP细胞对顺铂的敏感性，4 μg/mL顺铂处理后，未经转染处理的A549/DDP细胞的存活率为（84.2±9.8）%，而SIRT1 siRNA处理后的A549/DDP存活率较低，为（61.1±5.7）%，差异有统计学意义（*P* < 0.05），但转染无关siRNA序列不会影响A549/DDP细胞接受顺铂处理后的存活率（*P* > 0.05）（图 4A）。

**3:**
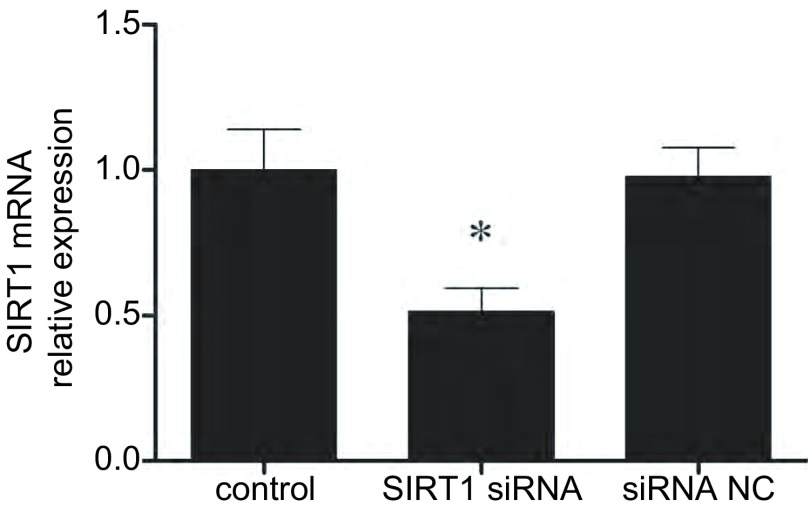
SIRT siRNA转染后，A549/DDP细胞的SIRT1 mRNA表达变化。*：与对照相比，*P* < 0.05。 The SIRT1 expression change caused by SIRT1 siRNA transfection. *: indicate *P* < 0.05 compared with the control group.

**4:**
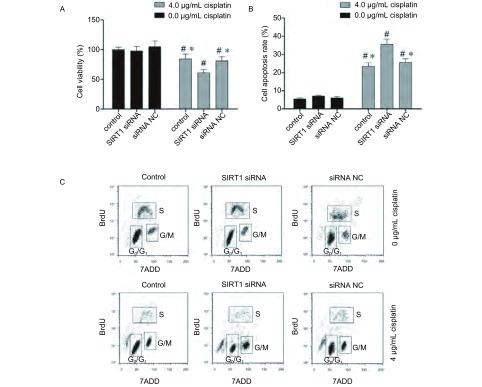
SIRT1 siRNA转染对A549/DDP细胞顺铂敏感性的影响。A：4 *μ*g/mL顺铂处理后各组细胞存活率；B：4 *μ*g/mL顺铂处理后各组细胞凋亡率；C：4 *μ*g/mL顺铂处理后各组细胞周期分布；*：与SIRT1 siRNA处理后的细胞相比比，*P* < 0.05；#：与未经顺铂处理的细胞相比，*P* < 0.05。 The effect of SIRT1 siRNA transfection on sensitivity of A549/DDP cells to cisplatin treatment. A: cell viability in each group after 4 *μ*g/mL cisplantin treatment; B: cell apoptosis rate in each group after 4 *μ*g/mL cisplantin treatment; C: cell cycle in each group after 4 *μ*g/mL cisplantin treatment; *: indicates *P* < 0.05 compared with SIRT1 siRNA treated cells; #: indicates *P* < 0.05 compared with cells without cisplatin treatment.

### 2.4 降低SIRT1表达可以增加顺铂造成的细胞凋亡

如图 4B所示，流式细胞仪分析可见，4 μg/mL顺铂处理后，未经SIRT1 siRNA转染处理的A549/DDP细胞凋亡率为（23.5±1.9）%，而SIRT1 siRNA处理后的A549/DDP细胞凋亡率增加，为（35.6±2.8）%，差异有统计学意义（*P* < 0.05）。转染无关siRNA序列不会影响A549/DDP细胞接受顺铂处理后的凋亡率（*P* > 0.05）。

### 2.5 降低SIRT1表达可以增加顺铂造成的G_2_期/M期阻滞

如图 4C所示，流式细胞仪分析结果显示，未经任何处理的A549/DDP细胞G_0_期/G_1_期和G_2_期/M期的百分比分别为（75.4±4.1）%和（8.1±0.5）%。4 μg/mL顺铂处理后，未经转染处理的A549/DDP细胞细胞G_0_期/G_1_期比例降至（60.4±6.3）%，G_2_期/M期比例升至（34.2±2.2）%，提示细胞发生了G_2_期/M期阻滞。SIRT1 siRNA处理后的A549/DDP细胞G_2_期/M期阻滞更为明显，其G_0_期/G期比例为（40.3±5.4%）%，G_2_期/M期比例为（50.2±6.4）%，以上差异均具有统计学意义（*P* < 0.05），转染无关siRNA序列不会影响A549/DDP细胞接受顺铂处理后的周期分布（*P* > 0.05）。

### 2.6 降低SIRT1表达可以增加Noxa表达

如图 5-图 6所示，SIRT1沉默后，A549/DDP细胞的Noxa表达显著提高，相对与未转染处理的细胞表达倍数为（1.65±0.12）（*P* < 0.05）。4 μg/mL顺铂处理可以轻微的增加A549/DDP细胞的Noxa表达，且SIRT1沉默后，顺铂引起的A549/DDP细胞的Noxa表达提高更为明显。该结果提示抑制SIRT1可以改善A549/DDP细胞对顺铂的敏感性，其机制可能为SIRT1抑制可以增加Noxa的表达。

**5:**
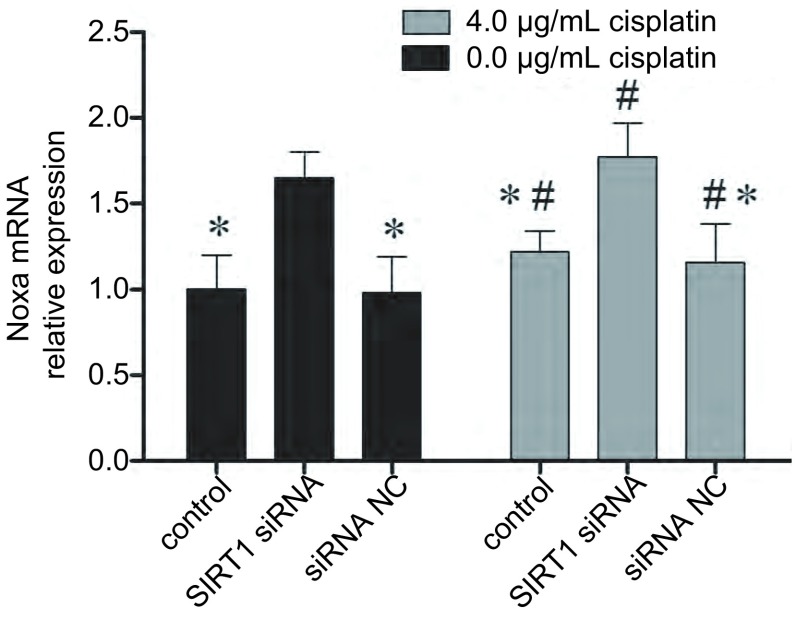
SIRT siRNA转染后，A549/DDP细胞的Noxa mRNA表达变化。*：与SIRT1 siRNA处理后的细胞相比，*P* < 0.05；#：与未经顺铂处理的细胞相比，*P* < 0.05。 The Noxa mRNA expression change caused by SIRT1 siRNA transfection. *: indicates *P* < 0.05 compared with control; #: indicates *P* < 0.05 compared with cells without cisplatin treatment.

**6:**
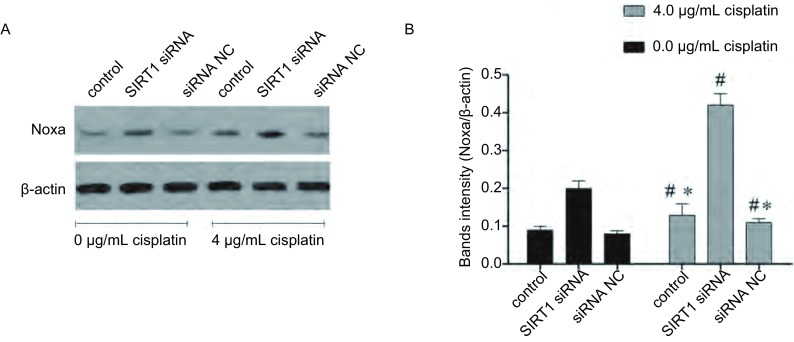
SIRT siRNA转染后，A549/DDP细胞的Noxa蛋白表达变化。A：各组Noxa蛋白条带；B：各组Noxa蛋白条带的灰度值；*：与SIRT1 siRNA处理后的细胞相比，*P* < 0.05；#：与未经顺铂处理的细胞相比，*P* < 0.05。 The Noxa protein expression change caused by SIRT1 siRNA transfection. A: Noxa protein bands images in each group; B: intensity of Noxa protein bands in each group; *: indicates *P* < 0.05 compared with control; #: indicates *P* < 0.05 compared with cells without cisplatin treatment.

## 3 讨论

NSCLC是最常见的呼吸系统恶性肿瘤，约占全部病例的80%。目前针对NSCLC的治疗主要包括手术、放疗及化疗，其中最为有效的治疗为外科手术^[8]^。然而由于诊疗水平的限制，特别是在发展中国家，不少患者在确诊时已经处于晚期，丧失了手术时机。这种情况下，非手术治疗显得尤为重要^[9]^。顺铂是广泛应用于NSCLC化疗方案中的药物，然而体内及体外研究均报道了NSCLC对顺铂敏感性降低的现象。不得不承认肿瘤细胞对顺铂敏感性较低或产生耐药性的机制是复杂的。任何一个涉及细胞生长发育、周期调控、凋亡、DNA损伤修复以及药物转运环节的异常均可能引起细胞对顺铂敏感性的降低^[10-12]^。本研究中我们发现SIRT1在顺铂耐药的A549/DDP细胞中存在高表达。使用siRNA降低A549/DDP细胞的SIRT1表达后，其对顺铂的敏感性提高。这些结果提示SIRT1可以影响A549细胞对顺铂的敏感性。实际上，既往的一些研究已经报道了SIRT1可以影响肿瘤细胞对化疗药物的敏感性。如Chen等^[13]^的研究发现SIRT1的过表达可以增加肿瘤的生长，降低肿瘤对索拉非尼的敏感性。Kojima等^[14]^发现在激素抵抗型前列腺癌PC3和DU145细胞株中，上调SIRT1表达可以导致细胞对化疗药物耐药。本研究和这些既往研究的结果一致。

至于SIRT1影响细胞对化疗药物敏感性的机制也有研究做出了报道。已有研究^[15]^发现使用RNA干扰技术降低SIRT1的表达后，P-糖蛋白和多药耐药蛋白表达降低，这两种蛋白均是公认的引起细胞化疗耐药的蛋白。还有研究^[16]^发现，向人胚肾细胞转染携带SIRT1序列的质粒后，细胞内与耐药相关的MDR1基因表达增高。这些研究提示SIRT1可能通过调节与耐药有关的一些基因或蛋白来影响肿瘤细胞对药物的敏感性。本研究发现，在NSCLC中SIRT1可能通过调节Noxa来影响细胞对顺铂的敏感性。Noxa具有明确的促凋亡作用，正如Baou等^[17]^的报道，硼替佐米可以提高Noxa的表达，进而诱导慢性淋巴细胞白血病细胞的凋亡。Noxa促进凋亡的机制之一与线粒体-细胞色素C途径有关，具体来说Noxa可以增加线粒体膜的通透性，促进细胞色素C的释放^[18]^。Noxa同时也是P53的下游基因，当细胞受到损伤刺激后，P53结合Noxa的上游启动序列后，可以增加Noxa的表达^[19]^。一定程度上讲，Noxa的促凋亡作用是依赖P53的，而P53是受SIRT1调控的，因此我们推测SIRT1可能以P53为桥梁，进而实现对Noxa表达的调控。当然，在一些情况系下，如低氧时，Noxa可不通过P53而得到激活，另外转录因子E2F1也可以直接激活Noxa，至于SIRT1是如何调控Noxa表达的还待于在未来研究中加以证实。
